# Is It Meanwhile Biomedical Sciences or Still “Ars Medica”?

**DOI:** 10.3390/cells9102313

**Published:** 2020-10-17

**Authors:** Wolfgang H. Jost

**Affiliations:** Parkinson-Klinik Ortenau, Center for Movement Disorders, 77709 Wolfach, Germany; w.jost@parkinson-klinik.de

Scientific work is usually quite time-intensive and frequently replete with frustrations. In the fields of mathematics and philosophy, for example, a single question might occupy one’s attention for an entire lifetime, without necessarily ever reaching a conclusion. Scientific work in the field of medicine evolves under essentially different conditions. Hippokrates therefore used the Greek term “iatrike techne” (ιατρική τέχνη) —or in Latin, “ars medica”—instead of medical science.

In medicine, we want and in fact have to reach conclusions quickly, partly because this is what we demand of ourselves to fulfill our criteria for scientific work, but also partly because we are searching for practical solutions which just cannot be delayed.

This is frequently the problem with translating basic science to clinical science. Basic scientists can very well focus preferably on concrete issues and demand concrete solutions, while clinical scientists must always live and work with limited knowledge and many uncertain factors for the simple reason that their studies cannot be conducted under laboratory conditions. We also must remember that we are involved with numerous premises and hypotheses which cannot be substantiated in the intermediate term, although they still determine our day-to-day work.

In order to be accepted, scientific hypotheses must be testable empirically. They are then either confirmed or refuted. In clinical settings, however, there is a considerable tendency towards generalizing, and from empirical and/or clinical findings, conclusions are drawn and hypotheses are advanced without testing for their validity. When results contradict the favored hypotheses (which means falsification), this very often leads to these results then being fully ignored. Our work is not consistently inductive or deductive. For example, we publish case reports and generalize relatively uncritically our observations and draw conclusions for the disease as a whole (induction), or we work deductively by drawing conclusions from the general picture of a disease (that is, the generic) regarding the concrete case (that is, the specific) (Figure 1). This situation takes on particular poignancy in Parkinsonism because Parkinsonism does not represent one single defined disease but rather a syndrome. When a disease is termed “idiopathic” (as is the case with the name idiopathic Parkinson’s syndrome), then work on the disease has to per se be conducted inductively. For example, to draw conclusions from a genetic form to an atypical form of Parkinson’s syndrome would be inaccurate, both inductively and deductively. This means that a symptom occurring in a patient with a genetic form does not necessarily have to also happen in all other Parkinson’s syndromes. This is compounded by the fact that the patients typically have other diseases and other medications as well, which makes evaluating their situation all the more difficult. The GBA (glucocerebrosidase) risk gene for Parkinson’s disease might be just such a case in point here: this gene is the proverbial “important puzzle piece” which however fails to complete the whole picture or to invalidate other results. The gene does offer additional new information which can contribute to further research, but, at the same time, it is not powerful enough to generate sweeping new hypotheses as to pathogenesis.

In a number of fields in clinical medicine, hypotheses build upon other hypotheses. However, even in the event that one hypothesis is disproven or is seen as no longer compelling and conclusive, the other related popular clinical hypotheses are not automatically rejected but rather continue to be accepted. For example, the Braak hypotheses are basically only reconcilable to a limited degree with other diagnostic and therapeutic routines. This all results in a rather complex situation which is interpreted by different workers in very different ways.

In the clinical sciences, we tend to view disease patterns much in the way of a puzzle: the more information we have (that is, the more puzzle pieces we have), the better we assume that we understand the entire disease. Vice versa, however, we also strongly want to form some concept of the bigger picture (even when our findings are still only insufficient) and might even be tempted to adapt our present findings accordingly: what does not match is made to match. Alternatively, at least when new findings do not fit our preliminary concept exactly, they are frequently ignored (because “what must not be, cannot be”).

With Parkinson’s syndrome, these aspects are particularly relevant in as much as the disorder has to be viewed multi-dimensionally. We not have only one single unified entity but rather many clusters which overlap considerably, congruent in the long run but still unique. Every new finding can essentially alter the basic concept.

It is a matter of course that every scientist considers their own publication work as highly important, while not forgetting to place it in the appropriate context with that of others. This is the theme of translational science, which calls for people who have schooling in both basic science and clinical medicine. They necessarily have to occupy themselves with the disease on both the clinical and the scientific level, but because the flood of information is constantly expanding, this is becoming less and less realizable: we just cannot know all the most recent studies. We have to satisfy ourselves with only a selection of the material (selective perception). The modern day reviewing process automatically means that certain opinions are better reinforced while others are relegated to the periphery.

When findings match the present day teachings and the corresponding publications are cited, they are more eagerly accepted than when the results contradict the mainstream. As a case in point, the literature on the role of the intestines in Parkinson’s syndrome was controversial in the 1990s and was accordingly viewed critically. In contrast, today, we face a large number of publications which directly postulate just such a role of the intestines and the microbiome in the pathogenesis of Parkinson’s, although, strictly speaking, the exact data do not (yet) substantiate this assumption.

These considerations form the basis for our publication “Molecular and Cellular Mechanisms of Parkinson’s Disease”. Workers from the field of the basic sciences and clinicians must cooperate; they must pool their findings and knowledge so as to develop a joint view. In this pursuit, there must be a willingness to accept the limits of everyone’s own research and to recognize one’s own errors. Too often, hypotheses that are simply inaccurate are perpetuated and become perennial sources of further research. We must be prepared to return to our original situation when a disease is first being described and then validate our hypotheses with our present day knowledge. In the present Special Issue, we wish to help resolve this problem by working together, pooling our efforts, and translating them into the biomedical sciences.

## Figures and Tables

**Figure 1:**
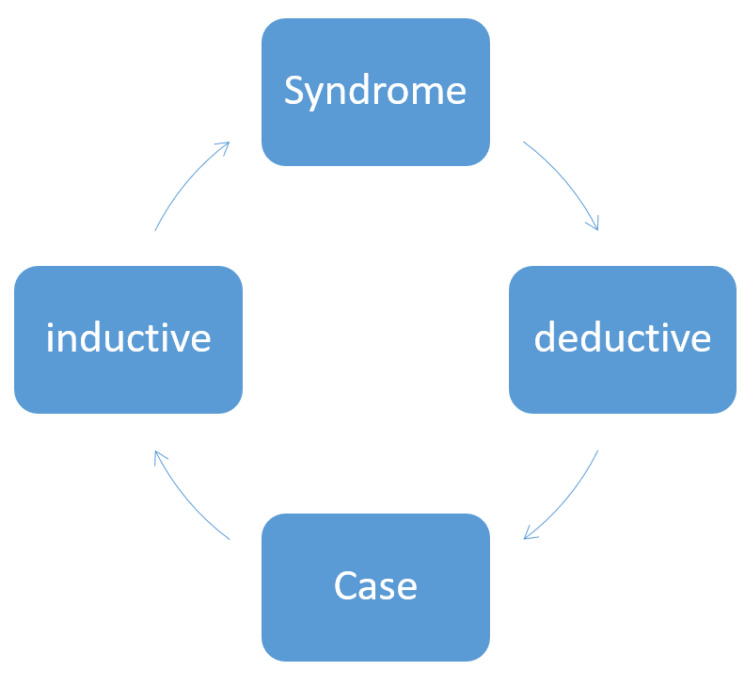
Deduction and induction in relation to syndromes (theory) and individual cases.

